# Pharmacological blockade of aquaporin-1 water channel by AqB013 restricts migration and invasiveness of colon cancer cells and prevents endothelial tube formation *in vitro*

**DOI:** 10.1186/s13046-016-0310-6

**Published:** 2016-02-24

**Authors:** Hilary S. Dorward, Alice Du, Maressa A. Bruhn, Joseph Wrin, Jinxin V. Pei, Andreas Evdokiou, Timothy J. Price, Andrea J. Yool, Jennifer E. Hardingham

**Affiliations:** Molecular Oncology, Basil Hetzel Institute, The Queen Elizabeth Hospital, Woodville, SA Australia; Discipline of Physiology, School of Medicine, University of Adelaide, Adelaide, SA Australia; Disciplines of Surgery and Orthopedics, School of Medicine, University of Adelaide, Adelaide, SA Australia; Medical Oncology, The Queen Elizabeth Hospital, Woodville, SA Australia; School of Medicine, University of Adelaide, Adelaide, SA Australia; Level 1, Basil Hetzel Institute, The Queen Elizabeth Hospital, 28 Woodville Road, Woodville, SA 5011 Australia

**Keywords:** Aquaporin 1, Inhibitor, Colon cancer, Migration, Invasion, Angiogenesis

## Abstract

**Background:**

Aquaporins (AQP) are water channel proteins that enable fluid fluxes across cell membranes, important for homeostasis of the tissue environment and for cell migration. AQP1 knockout mouse models of human cancers showed marked inhibition of tumor-induced angiogenesis, and in pre-clinical studies of colon adenocarcinomas, forced over-expression of AQP1 was shown to increase angiogenesis, invasion and metastasis. We have synthesized small molecule antagonists of AQP1. Our hypothesis is that inhibition of AQP1 will reduce migration and invasiveness of colon cancer cells, and the migration and tube-forming capacity of endothelial cells *in vitro*.

**Methods:**

Expression of AQP1 in cell lines was assessed by quantitative (q) PCR, western blot and immunofluorescence, while expression of AQP1 in human colon tumour tissue was assessed by immunohistochemistry. The effect of varying concentrations of the AQP1 inhibitor AqB013 was tested on human colon cancer cell lines expressing high versus low levels of AQP1, using wound closure (migration) assays, matrigel invasion assays, and proliferation assays. The effect of AqB013 on angiogenesis was tested using an endothelial cell tube-formation assay.

**Results:**

HT29 colon cancer cells with high AQP1 levels showed significant inhibition of migration compared to vehicle control of 27.9 % ± 2.6 % (*p* < 0.0001) and 41.2 % ± 2.7 (*p* <0.0001) treated with 160 or 320 μM AqB013 respectively, whereas there was no effect on migration of HCT-116 cells with low AQP1 expression. In an invasion assay, HT29 cells treated with 160 μM of AqB013, showed a 60.3 % ± 8.5 % decrease in invasion at 144 hours (*p* < 0.0001) and significantly decreased rate of invasion compared with the vehicle control (F-test, *p* = 0.001). Almost complete inhibition of endothelial tube formation (angiogenesis assay) was achieved at 80 μM AqB013 compared to vehicle control (*p* < 0.0001).

**Conclusion:**

These data provide good evidence for further testing of the inhibitor as a therapeutic agent in colon cancer.

**Electronic supplementary material:**

The online version of this article (doi:10.1186/s13046-016-0310-6) contains supplementary material, which is available to authorized users.

## Background

Colorectal cancer (CRC) is the third most commonly diagnosed cancer and the third leading cause of death resulting from cancer in the USA [1]. In Australia it is the second most commonly reported cancer diagnosis after prostate cancer, and the second leading cause of death after lung cancer [2]. A major determinant of patient prognosis is the stage at which the cancer is diagnosed, as surgery is considered curative in up to 70 % of early stage cases. Screening programs have helped with early diagnosis and intervention, but for those not participating in such programs some 12-25 % of CRC patients still present with advanced (stage IV) disease [3]. Furthermore, up to 30 % of patients diagnosed with early localised CRC (stage I or II) and up to 50 % with regional spread to lymph nodes or adjacent organs (stage III) eventually relapse with overt metastatic disease following ‘curative’ surgery [4]. Adjuvant chemotherapy is offered to stage III patients to eradicate potentially existing micro-metastases but the indication for such treatment in stage II disease is less certain where the benefit shown in clinical trials is small in absolute terms and thus the risk of fluorouracil toxicity likely outweighs the benefit [5]. The dilemma is finding a balance between the benefit of therapy, which may be incremental, and risk of harm, and to that end discovery of new therapeutic targets and pharmacological agents is a continuing goal for improving adjuvant cancer therapy.

### Aquaporins (AQP) and their role in cancer progression

Mammalian aquaporins are a family of 13 classes of intrinsic membrane proteins that assemble as tetramers (~30 kDa per subunit) and are known for their role in fluid homeostasis and trans-membrane transport of water and other small solutes [6, 7]. Specific classes of AQP channels have been implicated in enhanced migration, angiogenesis and metastasis in a variety of cancer types [8, 9], prompting the suggestion that inhibitors of AQP channels might provide new tools for cancer therapy [8]. The role of AQP1 in tumour migration and angiogenesis was first demonstrated in a murine melanoma tumour model: in AQP1 null mice tumours were smaller with fewer micro-vessels and more extensive necrosis as compared to AQP1 wild type mice, suggesting that AQP1 deletion impaired endothelial cell proliferation and angiogenesis [10]. AQP1 involvement in angiogenesis has been confirmed in other studies: in a murine melanoma tumour model, mice treated with AQP1 short interfering (si) RNA had significantly smaller tumours and lower microvessel and endothelial marker (factor VIII) densities compared to control mice, suggesting AQP1 knockdown impaired tumour growth and angiogenesis [11]. In mice with genetic deletion of AQP1, microvessel density was significantly reduced and also the number of lung metastases (5 ± 1/mouse) as compared with AQP1-expressing mice from the same genetic background (31 ± 8/mouse, *P* < 0.005) [12]. Colon tumour cells over-expressing AQP1 exhibited increased migratory and invasive capacity in wound healing (migration) and transwell invasion assays [13]. Over-expression of AQP1 in tumour cell lines resulted not only in a predicted increase in cell membrane water permeability, but also a 2 to 3-fold accelerated migration rate of the AQP1-expressing tumour cells as compared to control cells *in vitro*. AQP1 over-expression in mice increased the extravasation of tumour cells injected via the tail vein compared to control mice, and increased by 3-fold the number of lung metastases [14].

It is through enhanced water flux mediated by the AQP channels that cells are believed to acquire an enhanced migratory and invasive phenotype [13, 15]. Interestingly, AQP1 has been shown by our group to have dual water channel and gated ion channel functions [16, 17]. The AQP blocker AqB013, a derivative of bumetanide, has been characterised as a dose-dependent inhibitor of osmotic water fluxes mediated by mammalian AQP1 and AQP4 channels analysed in the *Xenopus laevis* expression system. AqB013 was shown to inhibit the AQP1 water channel function when applied extracellularly, and is thought to cross the membrane to occlude the water channel pore from the cytoplasmic side of the AQP1 channel [18]. Work here is the first to test the efficacy of AqB013 in inhibiting migration, invasion and angiogenesis in colon cancer cell line models.

## Methods

### Cell lines and cell culture

HT29 and HCT-116 colon cancer cell lines (ATCC, Manassas, USA) were cultured in complete medium composed of DMEM (Life Technologies, Carlsbad, CA, USA) supplemented with 1 x glutaMAX™ (Life Technologies), 1 x penicillin-streptomycin solution (Life Technologies) and 10 % foetal bovine serum (FBS). Cultures were maintained in 5 % CO_2_ at 37 °C. Their authenticity was confirmed (CellBank Australia, Melbourne, Vic). Human umbilical vein endothelial cells (HUVEC) (PromoCell, Heidelberg, Germany) were cultured in endothelial growth medium (PromoCell) according to the protocol supplied, and maintained in 5 % CO_2_ at 37 °C. Cells were confirmed to be negative for mycoplasma using the Universal Mycoplasma Detection kit (ATCC) according to the manufacturer's protocol.

### Colon tissue samples

Human colon tumour and matched normal mucosal tissue samples were obtained from 57 patients undergoing surgery for CRC at The Queen Elizabeth Hospital. The protocol was approved by The Queen Elizabeth Hospital Ethics of Human Research Committee (approval no. 1993059) and informed consent was obtained in all cases.

### Expression analysis of AQP1

#### Quantitative PCR

Cells at 70-80 % confluence were harvested and RNA extracted using the PureLink™ RNA Mini kit (Life Technologies). RNA was extracted from the frozen archived colon tumour and matched normal mucosa samples by pulverizing tissue under liquid nitrogen, and extracting RNA as before. RNA concentration was quantified using the NanoDrop 2000 spectrophotometer (Thermo Scientific, Waltham, MA, USA) and the integrity (RIN score) assessed using the 2100 Bioanalyzer (Agilent Technologies, Santa Clara, CA, USA). RNA (500 ng) was reverse transcribed using the iScript™ cDNA synthesis kit (Bio-rad, Carlsbad, CA, USA). qPCR of AQP1 and the reference gene phosphomannose mutase 1 (PMM1) [19] was performed using multiplex Taqman expression assays (Life Technologies), in triplicate via the CFX96™ Thermal Cycler (Bio-Rad). Each reaction contained 0.75 μL of each TaqMan® Gene Expression Assay (Life Technologies), 2 μL cDNA, 4.0 μL ultrapure water (Fisher Biotec, Wembley, WA, Australia) and 7.5 μL SsoFast™ probes supermix (Bio-rad) in a total volume of 15 μL. Results were calculated according to the 2^-ΔΔCt^ relative quantification method.

#### Western blot

Cells were lysed with RIPA buffer containing 1 % β-mercaptoethanol, 1 % HALT protease inhibitor 100X solution, 150 U Benzonase (all from Sigma, St Louis, MO, USA) on ice for 10 minutes, homogenized by passing through a 21 gauge needle and centrifuged at 14,000 x g for 15 minutes at 4 °C to pellet the cell debris. As AQP1 can be glycosylated [20], the supernatant was treated with PNGaseF (Promega, Madison, WI, USA) to cleave N-linked oligosaccharides. Protein was quantified (EZQ® assay, Life Technologies) and 50 μg of each sample was resolved by SDS-PAGE on a 12 % Mini-Protean® TGX Stain-Free™ Gels (Bio-Rad) and transferred to PVDF membranes using the Trans-Blot® Turbo™ Transfer Pack and System (Bio-Rad). Membranes were blocked with TBST containing 5 % skim milk for 1 hour and incubated overnight at 4 °C with anti-AQP1 rabbit polyclonal (H-55) (1/500; Santa Cruz Biotechnology Inc, Santa Cruz, CA, USA). Following three washes in TBST, membranes were incubated with goat anti-rabbit IgG HRP secondary antibody (1/2000) and Streptactin-HRP Conjugate (1/10000) (both from Bio-Rad) at room temperature for 1 hour, and washed. Chemiluminescence substrate was applied (Clarity™ Western ECL Blotting Substrate, Bio-Rad) and blots analysed using the ChemiDoc™ Touch Imaging System (Bio-Rad). Image Lab™ Software (Bio-Rad) was used for relative quantification of bands, normalized to total protein loaded in each lane.

#### Immunofluorescence

Cells grown on coverslips at 80 % confluence were fixed with 4 % paraformaldehyde and permeabilized with 0.5 % triton X-100. Image-iT® FX Signal Enhancer (Life Technologies) was applied directly to cover slips in accordance with manufacturer’s instructions. Cells were stained with a 1/400 dilution of rabbit polyclonal anti-human AQP1 (Abcam®, Cambridge, UK) and for the antibody isotype matched control, a 1/400 dilution of normal rabbit IgG was used (Cell Signaling Technology, Beverly, MA, USA). Cells were then stained with a 1/200 dilution of goat anti rabbit IgG H&L (Alexa Fluor® 568) secondary antibody (orange fluorescence) and the nuclei stained with NucBlue® Fixed Cell Ready Probes™ Reagent (blue fluorescence) (both from Life Technologies). Coverslips were mounted on slides with ProLong® Gold antifade reagent (Life Technologies), and images captured using the Zeiss LSM 700 microscope (Carl Zeiss Microscopy, Jena, Germany).

#### Immunohistochemistry

Tissue sections (5 μm) were deparaffinised by heating at 55-60 °C for 2 hours, soaking in xylene and hydrating by passing through a graded series of ethanol to water. Antigen retrieval was carried out by microwaving the slides in 10 mM sodium citrate for 20 mins. Endogenous peroxidase was quenched by incubating the slides in Peroxidazed I reagent (Biocare Medical, Concord, CA, USA) for 5 min and background staining was blocked by incubation in Background Sniper reagent (Biocare Medical). Slides were stained using a 1:100 dilution of AQP1 monoclonal antibody 10C11 (Abcam, Cambridge, UK) and detected using the MACH 3™ mouse HRP polymer detection system according to the manufacturer’s protocol (Biocare Medical). Slides were counterstained in haematoxylin (Sigma-Aldrich, St Louis, MO, USA).

### AQP1 inhibitor and vehicle control

AqB013 was dissolved in dimethyl sulfoxide (DMSO) at a 100 mM (stock solution), which was diluted into complete medium to yield the indicated working concentrations for the experiments. DMSO was used as the vehicle control at a dilution equivalent to that in the highest dose of the drug used in the treatments.

### Effect of AQP1 inhibition on cell migration (wound healing assay)

HT29 or HCT-116 cells were grown in complete medium (DMEM with 10 % FCS) to 80-90 % confluence in triplicate wells of a 24-well untreated plastic tissue culture plate, serum starved overnight, then treated with 1 μg/mL mitomycin C in complete medium to prevent cells from proliferating. Wells were treated with increasing amounts of the inhibitor with vehicle (DMSO) as control. A p10 pipette tip was used to scratch a wound through the cell monolayer and cells were monitored as they migrated across the wound using a Nikon Eclipse microscope (40X magnification) at time 0, 16, 32, 48, 64 and 80 hours: 20 measurements were taken of the wound width at each time point using the NIS-Elements software and averaged. Wound closure was calculated as a percentage relative to the initial wound width.

### Spheroid basement membrane extract (BME) invasion assay

Cells were cultured into spheres in an ultra-low attachment plate according to the manufacturer's protocol (Cultrex® 3D 96 Well Spheroid BME Cell Invasion Assay, Trevigen™), then covered with Invasion Matrix (a proprietary extracellular matrix blend comprised of basement membrane extract, derived from murine EHS sarcoma cells and collagen) together with the inhibitor AqB013 from 0 (untreated) to 160 μM, and with vehicle control. The area of the sphere was measured at each time point to assess invasion into the surrounding matrix, using the NIS-Elements software. The change in sphere area was calculated as a percentage relative to the initial sphere size; percent invasion of the test sphere was subtracted from the percent invasion of the vehicle control to indicate the extent of invasion into the matrix.

### Cell proliferation assay

HT29, HCT-116 or HUVEC were grown overnight, then AqB013 was added to triplicate wells from 0 (untreated) up to 320 μM. DMSO was added to triplicate wells as vehicle control. Proliferation was quantified after 48 hours using the Cell Titer 96® Aqueous Non- Radioactive cell Proliferation Assay (Promega) and absorbance at 490 nm read on a FLUOstar OPTIMA 96 well microplate reader (BMG Lab Tech, Ortenberg, Germany).

### Angiogenesis assay

Human umbilical vein endothelial cells (HUVECs) were seeded at 1 x 10^4^ cells per well in a 96 well plate pre-coated with BME (basement membrane extract: reconstituted protein matrix comprised of laminin, collagen IV, entactin, and heparin sulphate proteoglycan) and incubated in endothelial growth medium (PromoCell, Heidelberg, Germany) containing VEGF according to the Cultrex *In Vitro* Angiogenesis Assay Kit protocol (Trevigen, Gaithersburg, MD, USA). Wells were treated in triplicate with AqB013 from 0 (untreated) to 80 μM and with vehicle as a control. Cells were stained with Calcein AM dye at 24 hrs and imaged using a Nikon Eclipse fluorescence microscope. Endothelial tube formation was quantified as the number of junctions formed.

### Statistical analysis

Statistical analysis was carried out in Graph Pad® prism 5. A one-way ANOVA with Tukey’s multiple comparisons test was carried out for qRT-PCR results, for proliferation assays and for the angiogenesis assays. For the migration and invasion assays, a two-way ANOVA was performed to determine the significance between the data points of the final time point, also a first order polynomial regression was fitted to the data sets and an F-test used to determine significance (*p* < 0.05). Statistical significance was accepted at *p* < 0.05.

## Results

### Expression of AQP1

**Fig. 1:**
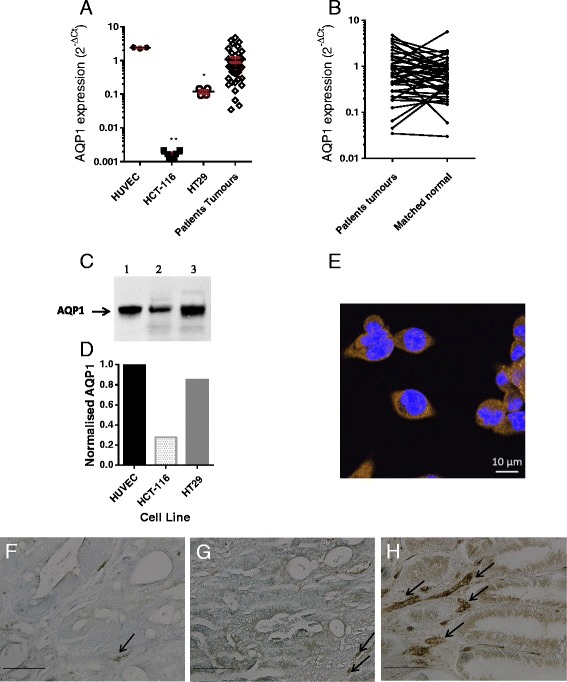
AQP1 expression. (**a**) relative expression of AQP1 (qPCR) in HUVEC, colon cancer cell lines, and human colon tumours (error bars show mean ± SEM; ** *p* = 0.004 ANOVA); (**b**) relative expression of AQP1 in CRC patients’ tumours compared to their matched normal mucosa. (**c**) western blot showing AQP1 monomer: lane 1 HUVEC, lane 2 HCT-116, lane 3 HT29. (**d**) corresponding quantification of bands using Image Lab™ Software (Bio-Rad); (**e**) immunofluorescence (IF) of HT29 colon cancer cells stained with anti-AQP1 and goat anti-rabbit secondary–Alexa 468 conjugate (orange) with NucBlue® stained nuclei overlay (63 x objective, scale bar = 10 μm). (**f**, **g**, **h**) immunohistochemistry of colon tumour sections staining for AQP1: F low, G moderate, H high expression. Arrows show examples of strong AQP1 staining of microvessels (20 x objective, scale bar = 0.1 mm)

Of the cell lines, the human umbilical vein endothelial cells (HUVEC) showed the highest expression of AQP1, HT29 showed a moderate level of expression, while HCT-116 showed a low level of expression. The range of expression levels was similar to that found in patients' tumour samples (Fig. 1a). Using the 2^-ΔΔCt^ relative quantification method, AQP1 was over-expressed (by >1.6-fold) in 22/57 (39 %) colon tumours compared to matched normal mucosa (Fig. 1b). Western blot (Fig. 1c) shows bands at approximately 28 KDa and relative quantification confirmed that AQP1 expression in HCT-116 cells was reduced by 67 % compared to HT29 cells, and by 72 % compared to HUVEC (Fig. 1d). Immunofluorescence staining showed both cytoplasmic and membrane AQP1 expression in HT29 cells (Fig. 1e). Immunohistochemistry of colon tumour sections showed that AQP1 is expressed variably in the apical membrane and in the cytoplasm. The range in staining for AQP1 concurs with the range of expression levels in tumour tissue at the mRNA level (Fig. 1a). In malignant crypt epithelial cells the expression of AQP1 varies from low intensity 1+ (Fig. 1f), moderate intensity 2+ (Fig. 1g) and high intensity 3 + (Fig. 1h): Endothelial cells of the micro-vessels show high staining intensity (arrows in Fig. 1 f–h) and reflects the high expression of AQP1 in endothelial cells at the mRNA level (HUVEC Fig. 1a).

### Treatment with AqB013 slows migration of colon cancer cells expressing significant amounts of AQP1

**Fig. 2:**
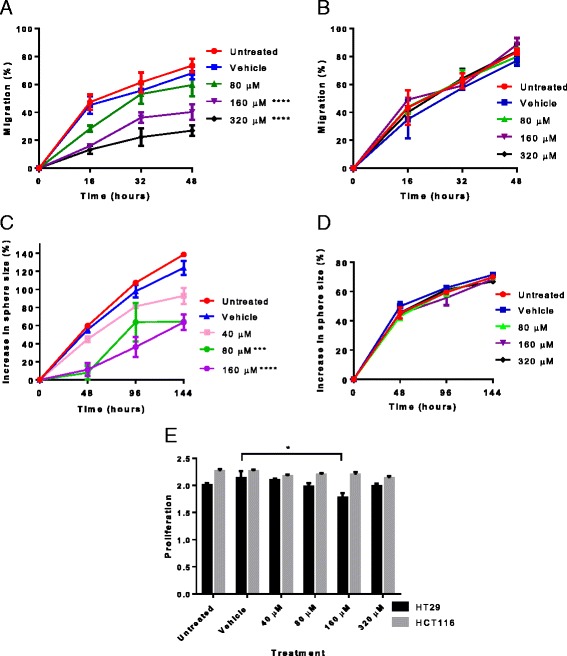
Migration and invasion. **a**, HT29 cells treated with 160 μM or 320 μM AqB013 had a significantly reduced migration compared to vehicle control. **b**, HCT-116 cells treated with up to 320 μM AqB013 showed no significant effect on wound closure. **c**, the effect of AqB013 on HT29 cell invasion (n = 3) measured by an increase in sphere size (%) relative to vehicle control: in spheres treated with 80 μM or 160 μM of AqB013 there was a significant decrease in invasion at 144 hours compared with vehicle. **d**, HCT-116 cells treated with up to 320 μM AqB013 (*n* = 3) showed no significant effect on invasion. ** *p* = 0.004; ***p < 0.001 ****p < 0.0001. **e**, AqB013 treatment up to 320 μM had no effect on cell proliferation of HCT-116, at 160 μM AqB013 HT29 showed 17% reduced proliferation (*p = 0.03 ANOVA). Proliferation measured in absorbance units at 490 nm. The error bars show standard error of the mean

At the final time-point HT29 cells treated with 160 μM or 320 μM AqB013 had a significantly reduced wound closure compared to vehicle control of 27.9 % ± 2.6 % (*p* < 0.0001) and 41.2 % ± 2.7 (*p* <0.0001) respectively (2-way ANOVA with Bonferroni post hoc test) (Fig. 2a). The dose response curve showed that the IC50 was 132.7 μM, Hill Slope -2.8. Treatment of HCT-116 cells (low level of AQP1 expression) with AqB013 showed no significant effect on migration (Fig. 2b).

### Treatment with AqB013 reduces invasion of colon cancer cells

The area of the spheres was measured as the cells invaded the surrounding matrix. For HT29 spheres treated with 80 μM or 160 μM of AqB013, there was a 59.5 % ± 1.4 % (*p* = 0.0003) or 60.3 % ± 8.5 % (*p* < 0.0001) decrease in invasion respectively at 144 hours compared to the vehicle control (Fig. 2c). A reduced rate of invasion was also found compared to the vehicle control (F-test, *p* = 0.001). The IC50 was 55.6 μM, Hill Slope -5.17. Treatment of HCT-116 with AqB013 showed no significant effect on spheroid invasion (Fig. 2d). There was no appreciable cytotoxic effect as there was no effect on proliferation of HCT-116 at concentrations of AqB013 up to 320 μM, although there was a small decrease in proliferation of HT29 treated at 160 μM AqB013 (Fig. 2e).

### Treatment of endothelial cells with AqB013 markedly inhibits tube formation

**Fig. 3:**
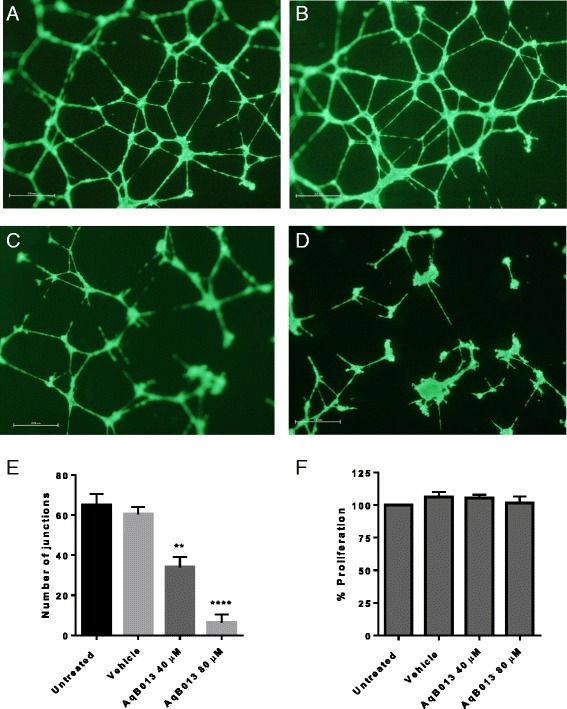
Angiogenesis assay. HUVEC tube-forming assay measured by the number of junctions: **a**, untreated HUVEC; **b**, vehicle treated HUVEC; **c**, HUVEC treated with 40 μM AqB013; **d**, HUVEC treated with 80 μM AqB013 (40 x magnification, scale bar = 0.5 mm); **e**, graph shows significant inhibition of endothelial tube formation by AqB013 at 40 μM and 80 μM, **p <0.01, ****p < 0.0001 respectively (ANOVA). **f**, AqB013 treatment had no effect on proliferation of HUVECs

Compared to vehicle control, treatment of HUVECs with 40 μM AqB013, showed a 43 % reduction in the number of junctions formed (34.0 ± 3.0, *p* < 0.01) while treatment with 80 μM AqB013 resulted in a reduction of 89.5 % (6.3 ± 2.4, *p* < 0.0001) (Fig. 3a–d). As expected there was no significant difference in the number of junctions formed between untreated and vehicle treated HUVECs (64.3 ± 3.0 versus 60 ± 2.5 respectively) (Fig. 3e). There was no effect on viability or proliferation of HUVEC treated at up to 80 μM AqB013 (Fig. 3f).

## Discussion

Small molecule pharmaceuticals have an established therapeutic use and our team has synthesised an AQP inhibitor, based on bumetanide [21], that blocks AQP1-mediated water flux [18]. Bumetanide is a loop diuretic that has long been used to treat patients with oedema [22]. Furthermore the incidence of clinically significant side-effects of bumetanide therapy is very low compared to that associated with chemotherapy drugs currently given as adjuvant therapy for CRC, making it an attractive alternative to current chemotherapeutics. AQP1 has been implicated in tumour progression in murine models and may thus serve as a potential target for small molecule inhibitors to treat cancer in subgroups expressing AQP1. The *in vitro* testing of drugs is the first step in establishing the efficacy of targeting specific molecules in abrogating the migration and invasion of cancer cells, or in suppressing angiogenesis.

In this study, inhibition of AQP1 by the inhibitor AqB013 was effective in reducing migration (wound closure assay) and invasion (spheroid formation assay) in the high AQP1-expressing HT29 cells, while not affecting migration or invasion in HCT-116 cells that had much lower expression of AQP1. As both untreated and vehicle-treated HT29 and HCT-116 cells showed similar efficiency of wound closure and invasion, these results suggest that AQP1 was indeed the target of inhibition. In breast cancer cells, AQP5 polarizes to the leading edge of migrating MDA-MB-231 cells, and that knockdown of AQP5 in these cells significantly suppressed cell migration velocity in narrow channels [23]. Similarly, knockdown of AQP5 in MCF7 breast cancer cells resulted in significantly reduced proliferation and migration [24]. However we have shown that the expression of AQP5 in HCT-116 is low (Additional file 1), similar to that of AQP1, suggesting that in these cells an alternative mechanism of migration is used which would explain why these cells are resistant to the inhibitory effect of AqB013. Migration in HCT-116 cells that express low amounts of AQP1 and AQP5 may be enhanced by expression of the calcium activated chloride channel TMEM16A as it has recently been reported that the high metastatic-potential colon cancer cell lines HCT-116 and SW620 express TMEM16A while primary colon cancer cell lines HCT8 and SW480 cells do not. Knockdown of TMEM16A by short hairpin RNA in SW620 resulted in significantly reduced migration in wound-closure assays [25]. In addition HCT-116 cells have been shown to have high levels of micro RNA 224 which has recently been shown to activate the Wnt-β catenin pathway to promote migration and invasion of HCT-116 cells [26, 27], rendering the cells resistant to the effects of AQP inhibition.

AQP1 has been shown to have dual water channel and gated ion channel functions [16, 28, 29]. The AQP1-mediated cationic conductance has been implicated in influencing rates of net fluid transport in primary cultures of choroid plexus [30], and similarly this mechanism may be responsible for regulating net fluid flux in migrating epithelial and endothelial cells. However work by the Yool group has previously shown that endogenous chloride conductance in *X. laevis* oocytes is not blocked by AqB013. Furthermore in mouse intact gastric antral muscle, the addition of AqB013 did not change the resting membrane potential and had no substantial effect on the rhythmic electrical conduction properties [18]. Taken together these data suggest that the effect of AqB013 on impeding the migration of human colon cancer cells and endothelial cells expressing AQP1 is mediated by blocking the water channel activity of AQP1.

## Conclusions

These studies have shown clear links between AQP1 activity and cancer cell migration and invasion, and endothelial cell tube–forming capacity, indicating the importance of characterising suitable AQP1 blockers. This study provides preliminary data showing that the AQP1 inhibitor AqB013 abrogates endothelial tube formation and reduces cancer cell migration and invasion and will be further investigated in an *in vivo* mouse xenotransplant model of human colon cancer. Small molecule pharmaceuticals have an established therapeutic use and as this new drug is a modification of bumetanide, it should be well-tolerated in cancer patients with far fewer side-effects than from currently used chemotherapeutic drugs. Furthermore, in view of the documented role of AQP1 in murine tumour angiogenesis [10, 12], it is envisaged that in metastatic CRC patients, AQP1 inhibitors may have a role combined with anti-vascular endothelial growth factor (VEGF) therapy, or as an alternative anti-angiogenesis therapy in cases that become resistant to anti-VEGF therapy. The inhibition of AQP1 clinically may slow down the progression of CRC, increasing the window for optimal treatment resulting in better survival outcomes, particularly in early stage cases where micro-metastatic disease is present. 

## Additional file

Additional file 1:
**Expression of AQP1 and AQP5.** qPCR (2^-ΔCt^) results normalised to reference gene PMM1. (PDF 95 kb)
