# Supplementary figures and images for: Pharmacological blockade of aquaporin-1 water channel by AqB013 restricts migration and invasiveness of colon cancer cells and prevents endothelial tube formation in vitro
Source: J Exp Clin Cancer Res. 2016 Feb 24;35:36. doi: 10.1186/s13046-016-0310-6 (PMC4765103; doi:10.1186/s13046-016-0310-6)

## qRT-PCR AQP1 and AQP5

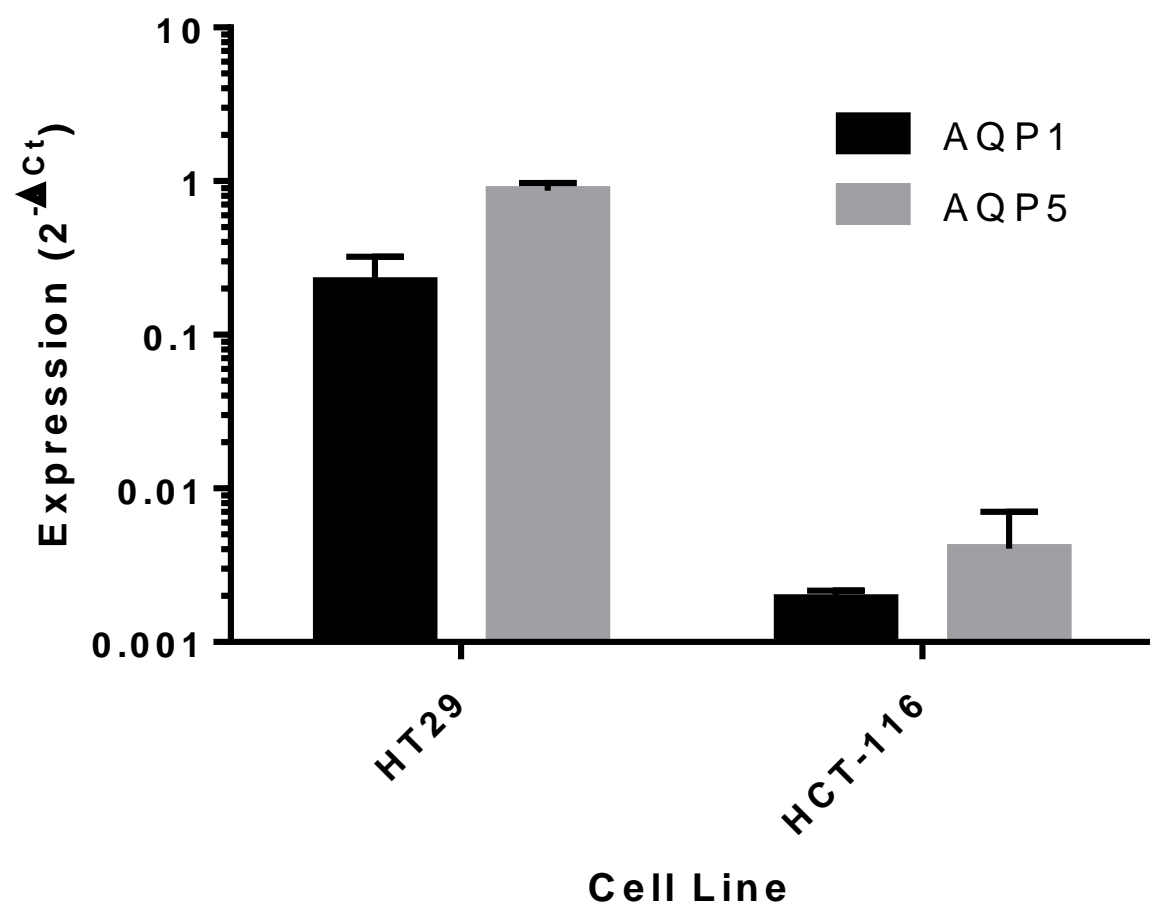

Supplement: Additional file 1: — Expression of AQP1 and AQP5. qPCR (2-ΔCt) results normalised to reference gene PMM1. (PDF 95 kb) [file 13046_2016_310_MOESM1_ESM.pdf]
